# Eye lens-derived Δ^14^C signatures validate extreme longevity in the deepwater scorpaenid blackbelly rosefish (*Helicolenus dactylopterus*)

**DOI:** 10.1038/s41598-023-34680-0

**Published:** 2023-05-08

**Authors:** Derek W. Chamberlin, Zachary A. Siders, Beverly K. Barnett, William F. Patterson

**Affiliations:** 1 Fisheries and Aquatic Sciences, University of Florida, 7922 NW 71st Street, Gainesville, FL 32611 USA; 2 Panama City Laboratory, Southeast Fisheries Science Center, National Marine Fisheries Service, 3500 Delwood Beach Road, Panama City, FL 32408 USA

**Keywords:** Fisheries, Population dynamics

## Abstract

Many members of the scorpaenid subfamily: Sebastinae (rockfishes and their relatives) exhibit slow growth and extreme longevity (> 100 y), thus are estimated to be vulnerable to overfishing. Blackbelly rosefish (*Helicolenus dactylopterus*) is a deepwater sebastine whose longevity estimates range widely, possibly owing to different regional levels of fisheries exploitation across its Atlantic Ocean range. However, age estimation has not been validated for this species and ageing for sebastines in general is uncertain. We performed age validation of northern Gulf of Mexico blackbelly rosefish via an application of the bomb radiocarbon chronometer which utilized eye lens cores instead of more traditional otolith cores as the source of birth year Δ^14^C signatures. The correspondence of eye lens core Δ^14^C with a regional reference series was tested with a novel Bayesian spline analysis, which revealed otolith opaque zone counts provide accurate age estimates. Maximum observed longevity was 90 y, with 17.5% of individuals aged to be > 50 y. Bayesian growth analysis, with estimated length-at-birth included as a prior, revealed blackbelly rosefish exhibit extremely slow growth (*k* = 0.08 y^−1^). Study results have important implications for the management of blackbelly rosefish stocks, as extreme longevity and slow growth imply low resilience to fishing pressure.

## Introduction

Bathybenthic and abyssal fishes have become increasingly exploited by fisheries in many regions of the world^1^. These deepwater species often have distinctive and complex life histories, exhibiting slow growth and extreme longevity, sometimes in excess of 100 years, and live in dark, cold, low-productivity habitats^1–3^. This combination of factors makes sustainable management of deepwater fisheries challenging, as they can be particularly susceptible to overfishing and slow to recover once overfished^4–6^. Catches can often be high as a fishery develops and a stock is fished down, but long-term sustainability often necessitates low catches else stocks are rapidly depleted below sustainable levels^7^. For example, orange roughy (*Hoplostethus atlanticus*), a deepwater trachichthyid harvested in the south Pacific, has a maximum sustainable yield estimated to be around 2% of unfished biomass given it is extremely long-lived (> 200 y) with low natural mortality^8^.

Age is a fundamental parameter in population ecology and fisheries science given accurate estimation of population dynamics rates relies on accurate and precise age estimates^9,10^. Ageing error most frequently materializes as an underestimate rather than an overestimate of true age, resulting in overestimates of growth and productivity^11^. Furthermore, underestimation of longevity, which is frequently used to estimate natural mortality (*M*), can result in the overestimation of *M*, thus overestimation of *F*_*MSY*_, the fishing mortality that produces maximum sustainable yield^12,13^. Biased age estimates can also propagate as uncertainty in stock assessment models, leading to overestimates of stock productivity and unsustainably high catch levels as management advice^14–17^. Therefore, accurate age estimates are vital to effective fisheries management.

Age in bony fishes is typically estimated by enumerating opaque zones in otoliths, with the assumption that opaque zone counts correspond to annual age^11^. Opaque zones are formed in otoliths due to seasonal differences in fish growth, which results in changes in the deposition of CaCO_3_ and protein on the otolith and alternating opaque and hyaline zones^18^. However, these zones are often difficult to distinguish in otolith sections of many deepwater species, which can result in biased ageing or low precision among readers^19,20^. Therefore, age validation is critical to accurate age estimation and effective management of deepwater fisheries^9^.

Blackbelly rosefish (*Helicolenus dactylopterus*), which is also known as bluemouth rockfish in part of its range, is a deepwater sebastine that is broadly distributed across the Atlantic Ocean, including the northern Gulf of Mexico (nGOM) and Mediterranean Sea^21^, and is commercially and recreationally exploited in U.S. waters and in commercial, multi-species longline fisheries in European Union waters^22,23^. However, there have been no stock assessments for blackbelly rosefish stocks in any part of its range since the early 2000s^24^, thus exploitation status is unknown. Previous age and growth studies in different global regions produced longevity estimates ranging widely from 10 to 43 y^22,25,26^, but ageing methods have yet to be validated for this species. Moreover, it is not uncommon for deepwater sebastines to have longevity estimates > 100 y^27^, implying a lack of resiliency to fishery exploitation^1,2^.

The bomb radiocarbon (^14^C) chronometer has been widely used to validate age estimation in marine fishes^11^, including many species in the nGOM^28–31^. This tool is based on the approximate doubling of atmospheric ^14^C in the 1950s and 1960s as a result of nuclear weapon testing, with atmospheric bomb ^14^C subsequently transferred into the ocean as dissolved inorganic carbon, or DIC^32,33^. The rise and subsequent decline in Δ^14^C, which is how ^14^C is typically measured and reported^34^, was incorporated into the aragonite skeletons of hermatypic corals around the world^35^, which have been used as reference time series for validating marine fish age estimates. In applying bomb ^14^C signatures for fish age validation, fish birth year Δ^14^C values, typically derived from otolith cores, are examined qualitatively or quantitatively for their correspondence to a regional Δ^14^C reference series^33,36^.

Successful applications of the bomb ^14^C chronometer for fish age validation have largely been restricted to species that spend their early life in shelf or epipelagic waters. This is due to the fact that Δ^14^C_DIC_ declines rapidly below a well-mixed surface layer^31,37^ and otolith C is largely (70–80%) derived from DIC^38^. Therefore, otolith cores of deepwater species are often depleted in ^14^C relative to surface waters^39–41^. The lack of correspondence of otolith core Δ^14^C values can lead to a conclusion that birth year, hence age, estimates are biased, when in fact the real issue is that Δ^14^C values in otolith cores reflect fossil C at depth instead of the bomb ^14^C signal from the well-mixed surface layer^31,42,43^.

Patterson et al.^44^ recently reported eye lens cores can be utilized as a source of birth year Δ^14^C, which may be especially beneficial for deepwater species because eye lenses are composed of metabolic C and nearly all organic C available to deepwater fishes is derived from epipelagic sources^45^. Additionally, eye lenses, like otoliths, are metabolically inert once formed and grow throughout a fish’s life^46,47^. Thus, eye lens cores may be an ideal source of birth year Δ^14^C for deepwater species^44^, and analysis of eye lens versus otolith core Δ^14^C values of nGOM deepwater (to 500 m) fishes suggests eye lens Δ^14^C does in fact match surface Δ^14^C_DIC_ values^48^. Therefore, the objectives of this study were to conduct age validation of blackbelly rosefish via application of the bomb ^14^C chronometer to eye lens Δ^14^C, as well as to estimate growth, longevity, and natural mortality from ageing data. Results are discussed in the context of blackbelly rosefish population dynamics, assessment, and management in the nGOM and other global regions where the species occurs.

## Methods

### Sample collection and age estimation

Fisheries-independent blackbelly rosefish samples were collected between 2015 and 2022 in the nGOM (28.55N to 29.39N and 86.50W to 88.02W) with hook and line at depths of 330 to 520 m. Animals were handled in accordance with the Guide for the Care and Use of Laboratory Animals under protocols approved by the University of Florida Institutional Animal Care and Use Committee (Protocol #202111559). Fish were labeled with zip-ties fastened through the buccal and opercular cavities and then placed on ice. Total length (TL) was measured to the nearest mm and each fish was weighed to the nearest g. Sex was determined by macroscopic examination of the gonads. Sagittal otoliths were removed, cleaned of adhering tissue, and stored dry in plastic cell wells. Both eyes were removed from the head, placed in plastic bags, and frozen at − 20 °C.

Fisheries-dependent samples were collected as part of the National Marine Fisheries Service Trip Interview Program (TIP). Port agents measured blackbelly rosefish to the nearest mm TL and the right and left sagittal otoliths were removed and stored dry in paper coin envelopes, and archived at National Marine Fisheries Service facility in Panama City, FL. Subsequently, all nGOM blackbelly rosefish otolith samples stored in the NMFS archives were shipped to the University of Florida Marine Fisheries Laboratory for age and growth analysis.

Left and right sagittal otoliths from fishery-dependent and -independent samples were weighed to the nearest mg. The left sagitta from each fish sample was embedded in epoxy and fixed to a glass slide with a toluene-based mounting medium. An approximately 0.4-mm thick transverse section was cut through the otolith core with a low-speed, diamond-bladed saw. Thin sections were affixed to glass slides with the mounting medium and the surface of the section was coated with the same medium to fill any cracks or abrasions from the sectioning process. Sections were viewed under a dissecting microscope with 25 × magnification and transmitted light to count opaque zones; for counts > 20, the edge of the otolith was observed under greater magnification (up to 60x). Opaque zones were enumerated independently by two experienced readers (WFP and DWC) without knowledge of morphometric data or the other reader’s opaque zone count. Timing of spawning and opaque zone formation in blackbelly rosefish otoliths is unknown, and consistent margin condition identification is difficult for long-lived fish due to compacted growth zones at the otolith margin; therefore, we only report integer age here. The index of average percent error (iAPE) was calculated between readers to assess between-reader precision of opaque zone counts^49^, and bias plots were constructed to examine trends in opaque zone counts between readers across ages^50^.

### Bomb ^14^C age validation

A subset of fisheries-independent blackbelly rosefish samples was selected for Δ^14^C analysis; fisheries-dependent samples were not considered because eye lenses were not available for those samples. All tools, aluminum foil, or vials that came in contact with otolith or eye lens samples were baked at 525 °C for 24 h to remove any residual C from the manufacturing process. Right otoliths were embedded in epoxy, fixed to a glass slide with mounting medium, and a 1.5-mm thick transverse section, centered on the otolith core, was cut with a diamond-bladed, low-speed saw. Sections were rinsed with 1% ultrapure nitric acid, repeatedly flooded with 18.3 ΜΩ cm^−1^ polished water, and, once dry, affixed to a glass slide with a toluene-based mounting medium. The core of the otolith was identified, and a trench was milled around the core using the automated milling capabilities of a New Wave Research Micromill fitted with a 0.5-mm diameter milling bit. Powdered aragonite generated from milling the trench was removed, leaving the core intact. The core of the otolith was then extracted by milling to a depth of 1.0 mm. The resulting material was weighed and stored in borosilicate glass vials.

The right eye lens of the blackbelly rosefish whose otoliths were cored were also cored for Δ^14^C analysis. Eyes were thawed and each lens was extracted through an incision made in the cornea. Each lens was then wrapped in aluminum foil and freeze dried for 12 h, which caused the outer laminae of the lens to split and begin flaking. Outer laminae were removed with forceps, targeting a core mass of approximately 0.7 mg. Once extracted, eye lens cores were stored in borosilicate glass vials.

Otolith and eye lens samples were analyzed for Δ^14^C and δ^13^C with accelerator mass spectrometry (AMS) at the National Ocean Sciences AMS (NOSAMS) facility at Woods Hole Oceanographic Institution. The resulting data are reported as Δ^14^C, which is the activity relative to the absolute international standard (base year 1950) corrected for δ^13^C fractionation and ^14^C age^34^. Blackbelly rosefish otolith and eye lens core Δ^14^C signatures and birth years were superimposed on the regional Δ^14^C reference series of coral^51–56^, and known-age red snapper otolith^30,56^ Δ^14^C values. Blackbelly rosefish birth year was estimated as year of collection—opaque zone count + 0.5 for each sample. Adding the fractional year accounts for the fact that the entirety of the otolith core from the primordium to the beginning of the first annulus was milled, and the target eye lens diameter of 0.7 mg is estimated to correspond to the first year of life as well. Given the mean birth date and the timing of opaque zone formation are not known for nGOM blackbelly rosefish, adding 0.5 y to the estimated birth year accounted for the fact eye lens and otolith core material were formed across some portion of the first year of life.

The fit of otolith core and eye lens core Δ^14^C signatures to the regional Δ^14^C reference series was initially evaluated visually to determine which corresponded best with the regional Δ^14^C reference series. Ageing bias was subsequently assessed by analyzing the eye lens core Δ^14^C signatures versus estimated birth years with a Bayesian spline model fit to the regional Δ^14^C reference series. The Bayesian spline model uses a penalized B-spline approach to fit a smooth function through the Δ^14^C reference series. The B-spline functions are continuous, piecewise polynomial functions (1 and 2) with the resulting smooth function coming from a linear combination of B-splines (3).

1 $$B_{j,k} \left( {x_{i} } \right): = \omega_{j,k} B_{{\left( {j,k - 1} \right)}} \left( {x_{i} } \right) + \left( {1 - \omega_{j + 1,k} } \right)B_{j + 1,k - 1} \left( {x_{i} } \right)$$

2 $$\omega_{j,k} : = \left\{ {\begin{array}{*{20}c} {\frac{{x_{i} - t_{j} }}{{t_{j + k - 1} - t_{j} }}} & {{\text{if}} t_{j} \ne t_{j + k - 1} } \\ 0 & {{\text{otherwise}}} \\ \end{array} } \right.$$

where ${B}_{j,k}\left({x}_{i}\right)$ is the B-spline function for the $j$’th member of a set of B-splines of order $k$ (the polynomial degree plus one) evaluated at a given birth year, ${x}_{i}$. Each B-spline function is defined as a linear combination of two B-splines with order $k-1$ with weights, ${\omega }_{j,k}$, defined at a given birth year value, ${x}_{i}$, for a given knot, ${t}_{j}$, from a sequence of knots ${\varvec{t}}={t}_{j}, \dots , {t}_{q}$. This sequence of knots, ${\varvec{t}}$, is extended, such that ${q}_{\text{extended}}=2*\left(k-1\right)+q$ to ensure that the B-spline functions cover the whole span of the knots. The set of B-splines of a given order, $k$, and sequence of knots, ${\varvec{t}}$, can be defined as:

3 $${{\varvec{S}}}_{k,t}\left({x}_{i}\right)=\left\{\begin{array}{cc}{\Sigma }_{j}{a}_{j}{B}_{j,k}\left({x}_{i}\right),& {a}_{j}\in {\mathbb{R}}\end{array}\right\}$$

where ${a}_{j}$ are the coefficients of the B-spline functions controlling the effect size of the B-spline. Put simply, the B-splines are a series of piecewise functions spanning the range of the birth years with weights that control how much each B-spline matters to each observed birth year. These B-splines are then multiplied by their coefficients, ${a}_{j}$, to fit the set of B-splines to the Δ^14^C reference series.

Choosing the number of knots can be done arbitrarily but can easily result in overfitting. To prevent overfitting and avoid tuning the number of knots, we chose an arbitrarily high level of knots, 50, and used a random-walk prior to control the smoothing (4).

4 $${a}_{1}\sim N\left(\mathrm{0,1}\right);{a}_{j}\sim N\left({a}_{j-1},\tau \right);\tau \sim N(\mathrm{0,1})$$

where a hyperprior is put on the B-spline coefficients, ${a}_{j}$, based on a random walk from the $j-1$ coefficient with standard normal priors for the first coefficient, ${a}_{1}$, and for the hyperprior standard deviation, $\tau$. The effect of this random-walk prior is that across a wide range knot numbers the resulting reference series fit is very similar. To assess the accuracy of age estimates, we fit the penalized B-spline to the reference series assuming the expected Δ^14^C reference series value came from a normal distribution (5).

5 $${Y}_{{\text{ref}},i}\sim N\left({\widehat{Y}}_{{\text{ref}},i},{\sigma }_{\text{ref}}\right);{\widehat{Y}}_{{\text{ref}},i}={{\varvec{S}}}_{k,t}({x}_{{\text{ref}},i})$$

where ${Y}_{{\text{ref}},i}$ is the observed Δ^14^C values, ${\widehat{Y}}_{{\text{ref}},i}$ is the expected Δ^14^C from the penalized B-splines using the observed reference series birth years ${x}_{{\text{ref}},i}$, and ${\sigma }_{\text{ref}}$ is the standard deviation of the observations around the expected values, the reference series process error. The set of penalized B-splines are then used to estimate the expected Δ^14^C value of the eye lens samples based on the observed birth year, ${x}_{{\text{obs}},i}$.

However, this does not account for systematic ageing bias so the observed birth years, ${x}_{{\text{obs}},i}$, are then adjusted and the expected Δ^14^C value re-estimated from the penalized B-splines to estimate the posterior of the ageing bias using (6):

6 $${Y}_{{\text{obs}},i}\sim N\left({\widehat{Y}}_{{\text{obs}},i},{\sigma }_{\text{obs}}\right);{\widehat{Y}}_{{\text{obs}},i}={{\varvec{S}}}_{k,t}({x}_{{\text{obs}},i}-\delta )$$

where ${Y}_{{\text{obs}},i}$ is the observed Δ^14^C values for the otolith and eye lens core samples, ${\widehat{Y}}_{{\text{obs}},i}$ is the expected Δ^14^C values for these samples, ${\sigma }_{\text{obs}}$ is the standard deviation of the observed Δ^14^C values around the expected (i.e., process error), and $\delta$ is the ageing bias. We assumed a uniformly flat prior on $\delta \sim U\left(-\infty ,\infty \right)$. We fit this model with the *cmdstanr* package^57^ in R^58^ using 8 chains, 1,000 warmup iterations per chain, and 125 sampling iterations per chain. The Gelman-Rubin statistic was used to assess chain convergence with values less than 1.1 indicating convergence^59^. The 95% Bayesian credible interval was computed for the birth year adjustment posterior distribution, and we assessed whether the estimated ageing bias was significantly different than zero via the probability of the maximum a posteriori (MAP) value was zero, with an inference of no bias if $p\left({\delta }_{\text{MAP}}=0\right)>0.05$.

Following age validation analysis, a linear regression was fitted to the otolith mass (mg) versus estimated age data. This included fishery-independent as well as fishery-dependent samples. Fishery-dependent samples could not be included in the eye lens-based age validation analysis since eye lenses had not been collected from those fish at the time of sampling. Therefore, computing the relationship between otolith mass and age estimates among all samples, including those from fishery-dependent samples, provided some means to assess the plausibility of longevity estimates.

### Growth and natural mortality estimation

A von Bertalanffy growth model (VBGM) incorporating length-at-birth^60^ as a prior was used to estimate blackbelly rosefish growth parameters (7)^61,62^.

7 $${\widehat{l}}_{t,j,VB}={L}_{\infty }-({L}_{\infty }-{L}_{0}){e}^{-k{t}_{j}}$$

where ${L}_{\infty }$ is the asymptotic length, ${L}_{0}$ is the length-at-birth, *k* is the Brody growth coefficient, and ${\widehat{l}}_{t,j,VB}$ is the predicted length at age $t$ derived from opaque zone counts for each otolith $j$. There were two opaque zone counts for each otolith section, so we assumed the observed age of each individual came from a Student’s T distribution with the true age as a latent parameter (8).

8 $$t_{j,r} \sim {\text{Student`s T}}\left( {\nu ,\hat{t}_{j} ,\sigma_{{{\text{obs}}}} } \right)$$

where ${t}_{j,r}$ is the observed age for otolith $j$ for reader $r$, $\nu$ is the degrees of freedom equal to the number of reads, ${\widehat{t}}_{j}$ is the true age, and ${\sigma }_{\text{obs}}$ is a measure of the between-reader error. This separates the ageing imprecision estimated by multiple readers of otolith samples from overall growth variability, ${\sigma }_{VB}$. We assumed that ${\widehat{l}}_{t,j,VB}$ came from a log-Normal distribution with standard deviation, ${\sigma }_{VB}$ (9).

9 $${l}_{t,VB}\sim \mathrm{log}N\left(\mathrm{log}\left({\widehat{l}}_{t,j,VB}\right),{\sigma }_{VB}\right)$$

where ${l}_{t,VB}$ is the observed lengths for each sample.

The VBGM was fit in a Bayesian framework in STAN using the *rstan* package^63^ in R^58^. Weakly informative priors were placed on ${L}_{\infty }$, *k*, ${\sigma }_{VB}$ using the predicted mean from a VBGM fit using maximum likelihood and setting the respective ${\sigma }_{\theta }$ as the mean parameter estimate times a 50% coefficient of variation. A literature search was conducted for the length-at-birth of blackbelly rosefish and an informative log-normal prior was placed on ${L}_{0}$ with a mean of 2.8 mm and a standard deviation of 1.4 mm^64^. The model was run over 8 chains with a warmup of 5,000 and 500 samples kept per chain. Models were checked for convergence visually with trace plots and by examining the Gelman-Rubin convergence diagnostic, with values < 1.10 indicating model convergence^59^.

Natural mortality was estimated using the Hamel and Cope^65^ estimator, which is based on the maximum observed age (10).

10 $$M=\frac{5.40}{{A}_{max}}$$

where A_max_ is the maximum observed longevity. Longevity based proxies have been shown to outperform proxies based on growth and environmental parameters^13^. Thus, mortality estimation was restricted to the Hamel and Cope^65^ method.

## Results

### Sample collection and age estimation

**Figure 1:**
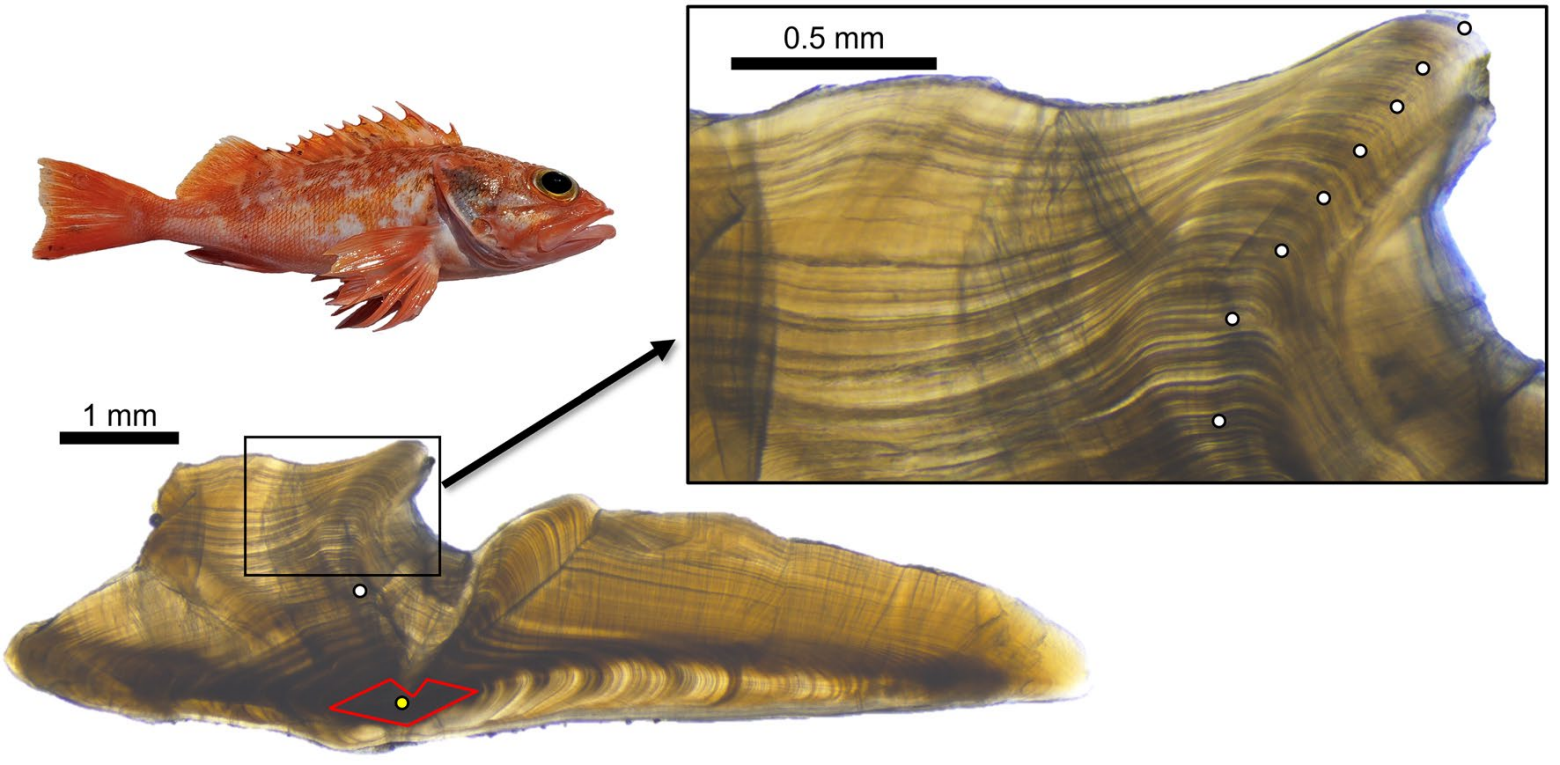
Digital image of a 351 mm total length blackbelly rosefish and a blackbelly rosefish otolith section from a 473 mm total length individual estimated to be 90 y by the primary reader. Yellow point denotes the otolith primordium. White points denote every 10th opaque zone, with the 20th through 90th opaque zones denoted in the inset. Red lines outline the approximate area targeted when milling the otolith core.

**Figure 2:**
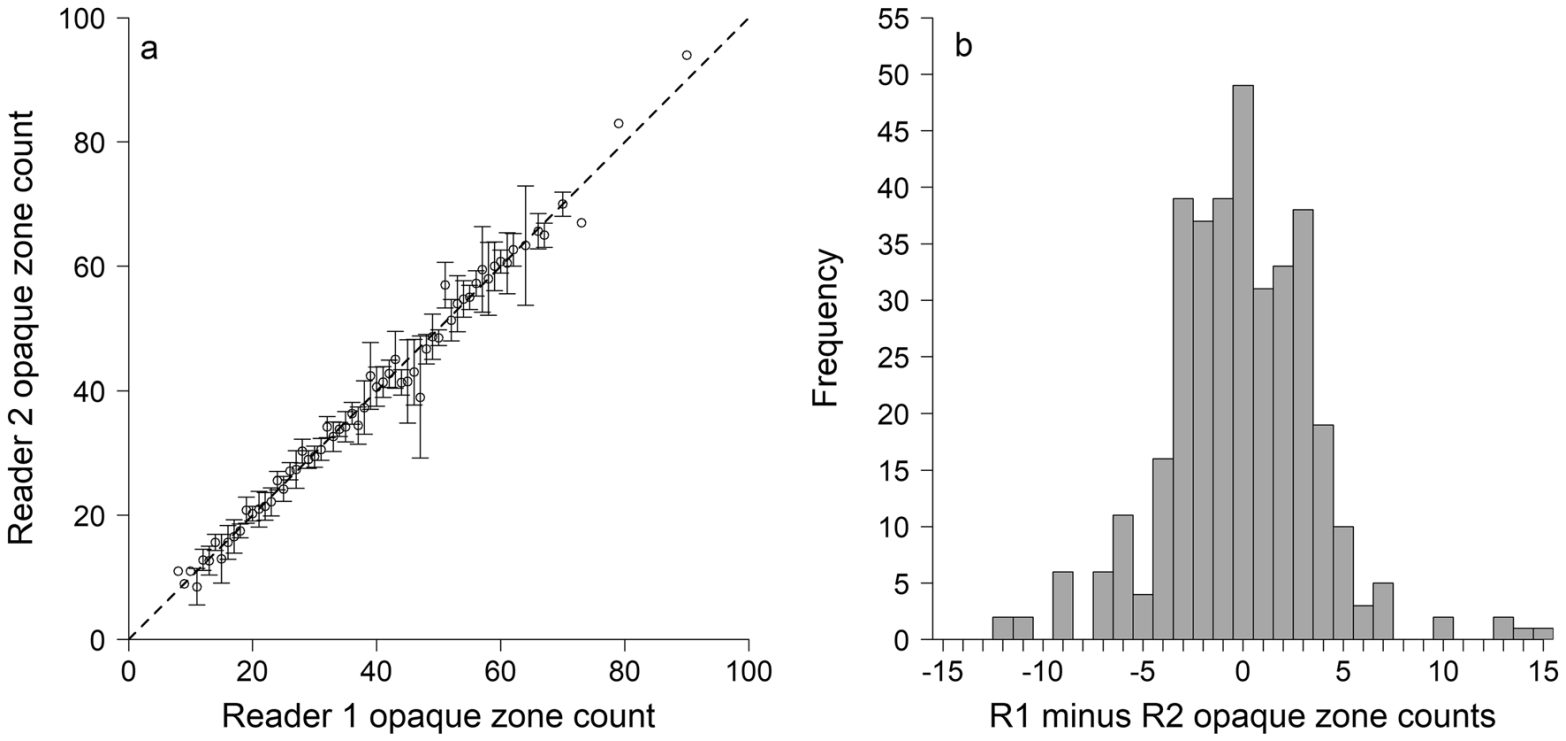
(**A**) Primary (WFP) and secondary (DWC) reader otolith opaque zone count comparison (n = 356). Error bars represent the 95% CI about the mean age assigned by reader 2 for all fish assigned a given age by reader 1. Dashed line denotes the 1:1 line of agreement. (**B**) Distribution of otolith opaque zone count differences between primary (R1) and secondary (R2) readers (n = 356).

In total, 356 nGOM blackbelly rosefish were sampled, consisting of 255 fisheries-dependent samples, and 101 fisheries-independent samples (Supplementary Fig. S1). Fisheries-independent samples were collected at depths of 338 to 520 m, with a mean depth of 377 m. Depth was not reported for fisheries-dependent samples. Otolith opaque zones were clear but become compacted after the 10th opaque zone (Fig. 1). Opaque zones were enumerated for each individual by both readers. There was no indication of systematic bias in opaque zone counts between reader 1 and reader 2 (Fig. 2) and the between-reader iAPE was 4.5%. Readers agreed on 13.7% of samples and 88.5% were within 5 opaque zones of each other. Otolith opaque zone counts ranged from 8 to 90 for reader 1 and 7 to 94 for reader 2.

### Bomb ^14^C age validation

**Figure 3:**
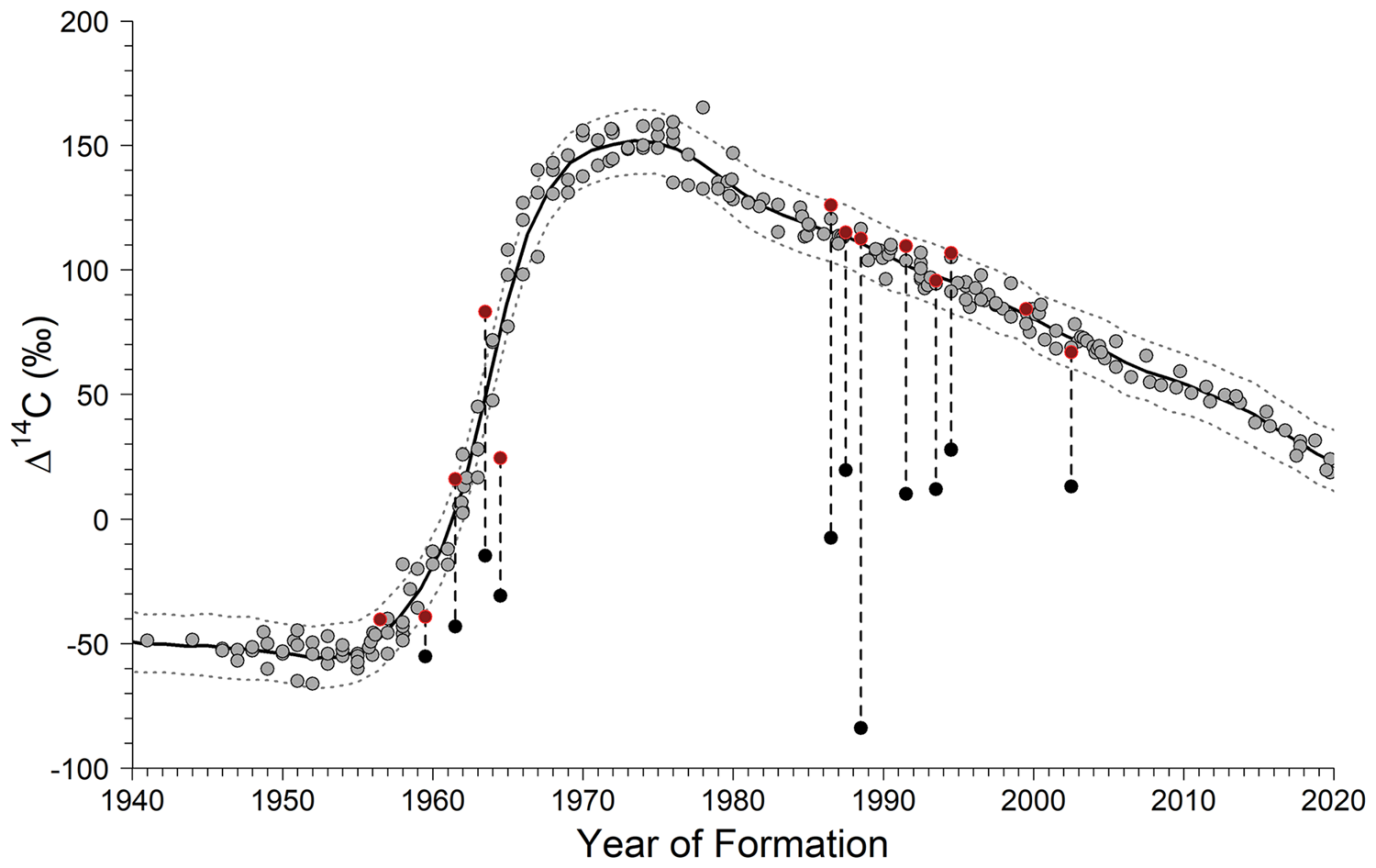
Blackbelly rosefish eye lens (n = 13, red circle) and otoliths core (n = 11, black circle) Δ^14^C versus the primary reader’s (WFP) year of formation (birth year) estimates overlaid on a regional coral and known-age otolith Δ^14^C reference series (1940–2021, gray circles). Dashed lines are 95% credible intervals.

**Figure 4:**
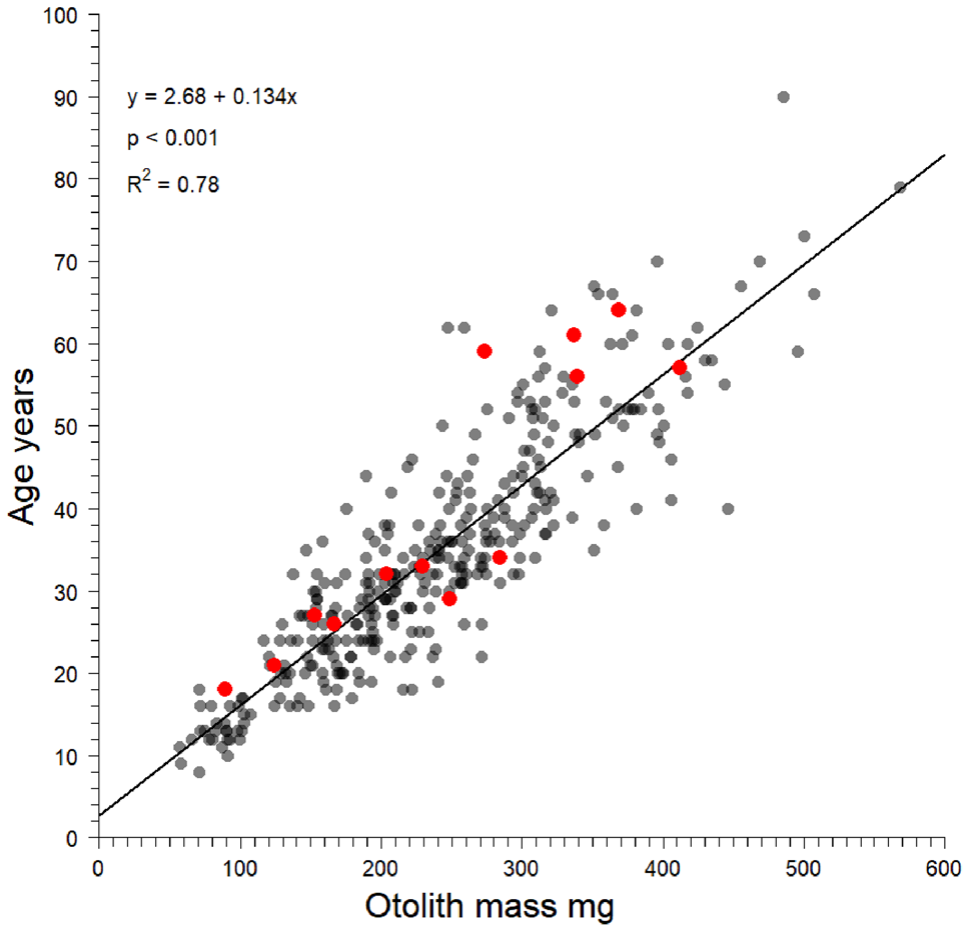
Primary reader age estimates versus blackbelly rosefish otolith mass (n = 356). Solid line is linear regression fit to the data. Red points are samples (n = 13) analyzed for eye lens-based Δ^14^C age validation.

Thirteen eye lens cores and eleven otolith cores were analyzed for Δ^14^C. Two otolith cores were broken and lost during processing, hence the mismatch between sample sizes. Otolith opaque zone counts for validation samples ranged from 15 to 63, with resulting birth year estimates ranging from 1956.5 to 2002.5 (Table 1). Otolith cores were considerably depleted in ^14^C relative to the regional reference series, while the eye lens core Δ^14^C signatures corresponded well with the reference series (Fig. 3). Thus, age validation analysis was restricted to eye lens core Δ^14^C. Results of the Bayesian validation analysis ($p$ = 0.50) demonstrated otolith opaque zone counts provided accurate age estimates for blackbelly rosefish (Fig. 3 and Supplementary Figs. S2 and S3). The Gelman-Rubin convergence diagnostic (1.01 for all parameters) and trace plots indicated the model converged (Supplementary Figs. S2 and S3). There was a significant relationship between otolith mass and age (*p* < 0.001; R^2^ = 0.78; Fig. 4), and the residuals were evenly distributed about the regression.

**Table 1:** Blackbelly rosefish eye lens and otolith core samples analyzed for Δ^14^C with accelerator mass spectrometry, along with otolith-derived age and birth year estimates.

Collection year	Age	Birth year	Eye lens NOSAMS#	Lens core mass mg	Lens core Δ^14^C ‰	Lens core σ	Lens core δ^13^C ‰	Otolith NOSAMS#	Otolith core mass mg	Otolith core Δ^14^C ‰	Otolith core σ	Otolith core δ^13^C ‰
2020	18	2002.5	OS-157986	0.82	67.00	2.3	− 19.05	OS-157937	1.46	13.21	3.2	− 4.60
2020	21	1999.5	OS-156627	0.66	84.24	2.1	− 18.96	*	*	*	*	*
2020	61	1959.5	OS-156628	0.83	− 39.08	2.6	− 17.23	OS-156832	1.79	− 55.12	2.6	− 4.88
2020	26	1994.5	OS-156629	0.53	106.80	2.9	− 18.46	OS-156833	1.72	27.90	2.9	− 5.11
2020	64	1956.5	OS-157987	0.70	− 40.16	2.2	− 17.88	*	*	*	*	*
2020	29	1991.5	OS-156630	0.77	109.68	2.2	− 18.86	OS-156834	2.27	10.30	2.5	− 4.47
2020	56	1964.5	OS-156631	0.76	24.66	2.4	− 18.02	OS-156910	1.73	− 30.78	3.0	− 4.82
2020	57	1963.5	OS-156633	0.69	83.18	2.4	− 18.19	OS-156913	2.42	− 14.55	2.8	− 4.72
2020	34	1986.5	OS-156634	0.44	125.97	2.9	− 18.23	OS-156914	1.54	− 7.39	2.9	− 4.61
2020	32	1988.5	OS-157988	0.84	112.61	2.8	− 17.84	OS-157938	2.50	− 83.88	2.3	− 4.29
2020	33	1987.5	OS-157989	0.83	115.09	2.9	− 18.10	OS-157939	2.04	19.80	2.6	− 4.63
2020	27	1993.5	OS-156690	0.90	95.78	2.3	− 18.31	OS-156919	1.41	12.06	3.4	− 4.79
2020	59	1961.5	OS-157975	0.97	16.14	2.4	− 17.65	OS-157935	2.34	− 43.02	2.3	− 5.04

σ is the measurement error for AMS analysis of Δ^14^C. * = sample lost prior to analysis.

### Growth and natural mortality estimation

**Figure 5:**
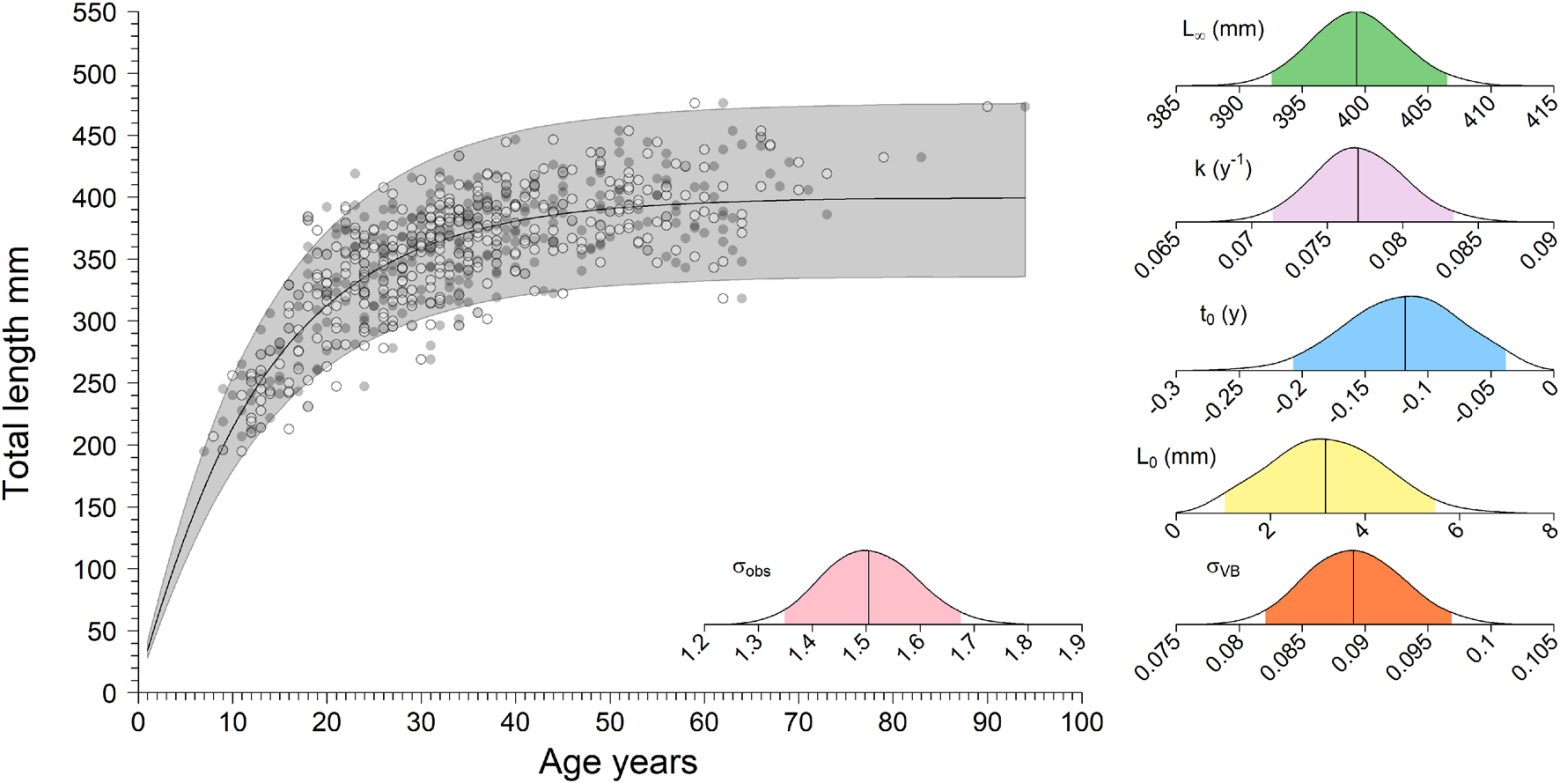
von Bertalanffy growth model (VBGM) fitted to blackbelly rosefish age (y) and total length (mm) data. White points with a black border are the primary reader’s (WFP) age estimates and gray points are the secondary reader’s (DWC) age estimates. The median (black solid line) and 95% credible intervals (CI; shaded area) are shown for the model-predicted length at age t (*l*_*t*_) from age-0 to age-94. Posterior distributions and 95% CI (shaded area) of VBGM parameter estimates are shown for asymptotic length (${L}_{\infty }$), Brody growth coefficient (*k*), the theoretical age at which length is 0 (*t*_0_), length-at-birth (${L}_{0}$), the measure of inter-reader error (${\upsigma }_{\text{obs}}$), and the likelihood standard deviation of the VBGM (${\upsigma }_{VB}$).

The Bayesian VBGM modeling produced a median growth coefficient (*k*) estimate of 0.08 y^−1^. The median *L*_*∞*_ estimate was 399.8 mm, while *t*_0_ was estimated to be − 0.10 y, and *L*_0_ was 2.95 mm (Table 2, Fig. 5). The Gelman-Rubin convergence diagnostic (< 1.1) and trace plots indicated the model converged (Table 2, Supplementary Fig. S4). Natural mortality was estimated at 0.06 y^−1^ with the Hamel and Cope^65^ method, based on maximum observed longevity of 90 y.

**Table 2:** Prior, posterior median, and 95% credible intervals (CI) for blackbelly rosefish von Bertalanffy growth model (VBGM) parameters and derived parameters.

Parameter	Prior	Posterior median (95% CI)	$\widehat{R}$
${L}_{\infty }$(mm)	${L}_{\infty }$~ HN(402.5, 201.3)	399.8 (392.5–407.7)	1.00
*L*_0_ (mm)	${L}_{0}$~ HN(2.80, 1.40)*	2.95 (0.50–5.66)	1.00
*k* (y^−1^)	$logit(k)$~ N(0.074,0.037)	0.076 (0.071–0.081)	1.00
${\upsigma }_{obs}$	${\upsigma }_{obs}$~ HN(31.49, 15.74)	1.50 (1.35–1.67)	1.00
${\upsigma }_{VB}$	${\upsigma }_{VB}$~ HN(31.49, 15.74)	0.088 (0.082–0.096)	1.00
*t*_0_	–	− 0.10 (− 0.19– − 0.02)	–

${L}_{\infty }$ is the asymptotic length, *L*_0_ is the length at birth, *k* is the Brody growth coefficient, ${\upsigma }_{\text{obs}}$ is the measure of inter-reader error, ${\upsigma }_{VB}$ is the likelihood standard deviation of the VBGM, and *t*_0_ is the theoretical age at length zero. $\widehat{R}$ is the Gelman-Rubin convergence diagnostic.

*Informed by Moser et al.^64^.

## Discussion

Results of eye lens core Δ^14^C analysis validate blackbelly rosefish otolith opaque zone counts as accurate estimates of age. The observed maximum longevity of 90 y indicates extreme longevity in this species, with correspondingly low natural mortality (*M* = 0.06 y^−1^), suggesting blackbelly rosefish stocks may be highly vulnerable to overfishing. Study results also provide a template to validate age estimates of other deep-water marine fishes, which are increasingly exploited^1^. There is a close relationship between *M* and the fishing mortality that produces the maximum sustainable yield (${F}_{MSY}$)^12^. Therefore, the low level of *M* for blackbelly rosefish suggests their populations can only sustain minimal fishing mortality, and stocks may not be able to support substantial targeted fisheries long-term. There is not a directed fishery currently for this species in the nGOM (e.g., annual landings of 3.5 metric tons between 2000 and 2021). Nonetheless, its life history suggests conservative management would be prudent there, and especially in global regions where blackbelly rosefish are directly targeted.

In the U.S., blackbelly rosefish are harvested recreationally and commercially in the nGOM and in the Atlantic Ocean, although the fishery in the nGOM is basically a bycatch fishery. As a result, the nGOM stock is not heavily exploited, averaging approximately 3.5 mt landed per year since 2000. Conversely, the Atlantic stock has experienced a rapid increase in exploitation since 2000, particularly in the recreational sector. Landings have increased from approximately 6 mt in 2000 to 115 mt in 2021, an 1800% increase. The US Atlantic stock is currently unassessed, but the composition of Atlantic landings sampled in the 1990s had a max age of 30 years, and a much more truncated age distribution than we report for the nGOM^22^. We cannot evaluate whether recent landings levels in the US Atlantic catch exceed MSY, but the data presented here could be informative for a data-limited assessment of that stock.

Blackbelly rosefish are also exploited but unassessed in the Portuguese Azores and the Mediterranean Sea^23,66^. Landings estimates in the multispecies bottom longline fishery in the Azores average approximately 350 mt of blackbelly rosefish per year, with over 11,500 mt in total landed between 1985 and 2018^23^. The smaller Mediterranean fishery landed just under 50 mt in 2003 in Catalonia alone^66^. The low level of natural mortality estimated for GOM fish suggests only low levels of fishing mortality are likely to be sustainable for these stocks. The rapid increase in recreational landings in the U.S. Atlantic and the sustained landings in the Azores and Mediterranean underlie the need for stock assessments to estimate stock status and prevent overfishing.

Accurately estimating age and growth are critical for effective stock assessment and management^67^. However, doing so often requires robust sample sizes that are spread across the full age range of the stock^68,69^. Size-selectivity in which smaller individuals are less vulnerable to capture can result in the underestimation of the theoretical age at which length is equal to zero (*t*_0_) and the Brody growth coefficient (*k*), which is related to stock productivity^69^. The age and length data presented here clearly show a gear bias as no fish smaller than 195 mm or younger than 8 y were captured. Fitting a traditional von Bertalanffy growth function would have resulted in biased parameter estimates, appearing to show slower growth, thus even lower productivity than estimated here^69^. The use of the Bayesian model that incorporated a size-at-birth prior to fitting the VBGM to validated age estimates produced a realistic fit to the data without having to fix parameter estimates.

The Brody growth coefficient in the blackbelly rosefish VBGM was estimated at 0.08 y^−1^, which is comparable to the slow growing, deep-water orange roughy (*Hoplostethus atlanticus*) in the southwest Pacific^70,71^ and the sebastine thornyheads (*Sebastolobus altivelis* and *S. alascanus*) in the northwest Pacific^72^. The orange roughy fisheries in New Zealand and Australia are notorious for their collapse in the 1970s and 1980s due to age misspecification and resultant overexploitation^11,73^. The similarity in growth rates between blackbelly rosefish and orange roughy may suggest blackbelly rosefish stocks are similarly vulnerable to overexploitation^1^, thus also indicates conservative management approaches are warranted.

Deepwater marine fishes are often difficult to age and many are long-lived^27,74^. There have been attempts to validate age estimates of deepwater fishes via radiometric methods^74,75^, as well as application of the bomb ^14^C chronometer, using otolith-derived birth year material^39,76,77^. However, these attempts have often served to increase rather than decrease ageing uncertainty. Radiometric age estimates tend to have high levels of uncertainty, with the thornyhead rockfishes (*Sebastolobus alascanus* and *S. altivelis*) in the northwest Pacific having mean radiochemical age uncertainty of ± 7.9 y, based on analytical uncertainty^1,72^. Notably, this uncertainty increases with age, with a mean radiochemical age uncertainty of ± 16.4 y for samples estimated to be older than 40 y. Therefore, while radiometric methods can provide evidence of extreme longevity in long-lived fishes, measurement error means actual age estimates are highly uncertain.

Application of the bomb ^14^C chronometer to otolith (birth year) Δ^14^C signatures also has been similarly uninformative for age validation of long-lived, deepwater marine fishes, albeit for other reasons than radiometric ageing. This uncertainty is due to the fact that otolith cores are often substantially depleted in ^14^C relative to reference series that index Δ^14^C_DIC_ in the well-mixed surface layer^39,76–78^. This trend has also been observed in long-lived sebastines in the north Pacific Ocean, Gulf of Alaska, and Bering Sea^41,79^, as well as other *Helicolenus* spp. in the western Pacific^40^. The offset between surface reference and otolith core Δ^14^C for species living at depths > 500 m is often reported to be 100–150‰, which is in the same range as the offset between nGOM blackbelly rosefish otolith core Δ^14^C and reference series Δ^14^C values. However, data reported here, as well as for the suite of nGOM deepwater fishes^48^, suggests deriving birth year Δ^14^C signatures from eye lens rather than otolith cores may enable the bomb ^14^C chronometer to be generally effective for age validation of deepwater fishes. It is currently unclear, however, if this approach has some depth threshold to its effectiveness, but to date it has been successfully applied to upper slope reef fishes that live at depths as great as 500 m. Given vertical migrants routinely (daily) transit from depths of 1,000 m or more into the epipelagic to forage^80–82^, it may be that the organic carbon available at those depths also has a contemporary surface bomb ^14^C signature. However, further testing, and perhaps coupling eye lens Δ^14^C signatures with either radiometric ageing or amino acid racemization^83^ approaches, could resolve this question.

## Supplementary Information

Supplementary Figures.

## Data Availability

The data underlying this article are provided within the published article or will be shared on reasonable request to the corresponding author.
