# Supplementary material for: Eye lens-derived Δ14C signatures validate extreme longevity in the deepwater scorpaenid blackbelly rosefish (Helicolenus dactylopterus)
Source: Sci Rep. 2023 May 8;13:7438. doi: 10.1038/s41598-023-34680-0 (PMC10167307; doi:10.1038/s41598-023-34680-0)
Supplement: Supplementary file 1 — Supplementary Figures. [file 41598_2023_34680_MOESM1_ESM.pdf]

Eye lens-derived $\Delta^{14}C$ signatures validate extreme longevity in the deepwater scorpaenid blackbelly rosefish (*Helicolenus dactylopterus*)

Derek W. Chamberlin[a*], Zachary A. Siders[a], Beverly K. Barnett[b], and William F. Patterson III[a]

[a]University of Florida, Fisheries and Aquatic Sciences, 7922 NW 71st Street, Gainesville, FL 32611, USA

[b]National Marine Fisheries Service, Southeast Fisheries Science Center, Panama City Laboratory, 3500 Delwood Beach Road, Panama City, FL 32408, USA

*Corresponding Author; derek.chamberlin@ufl.edu

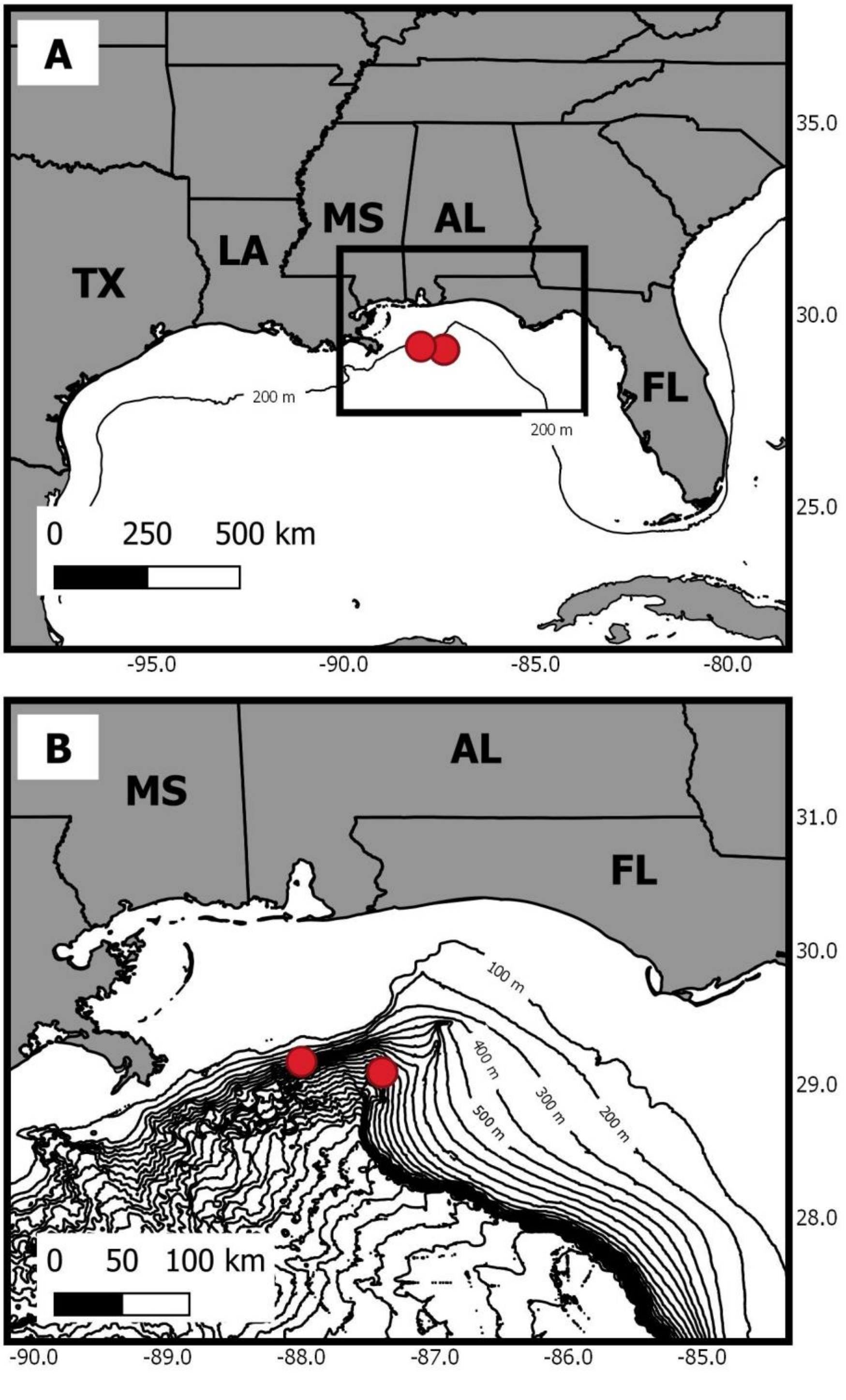

Figure S1. A) Locations of sample sites where fish sampled for ^{14}C were collected, fisheries-dependent samples were landed in Florida (FL) and Louisiana (LA) and B) location of ^{14}C fish sampling sites with 100m contours.

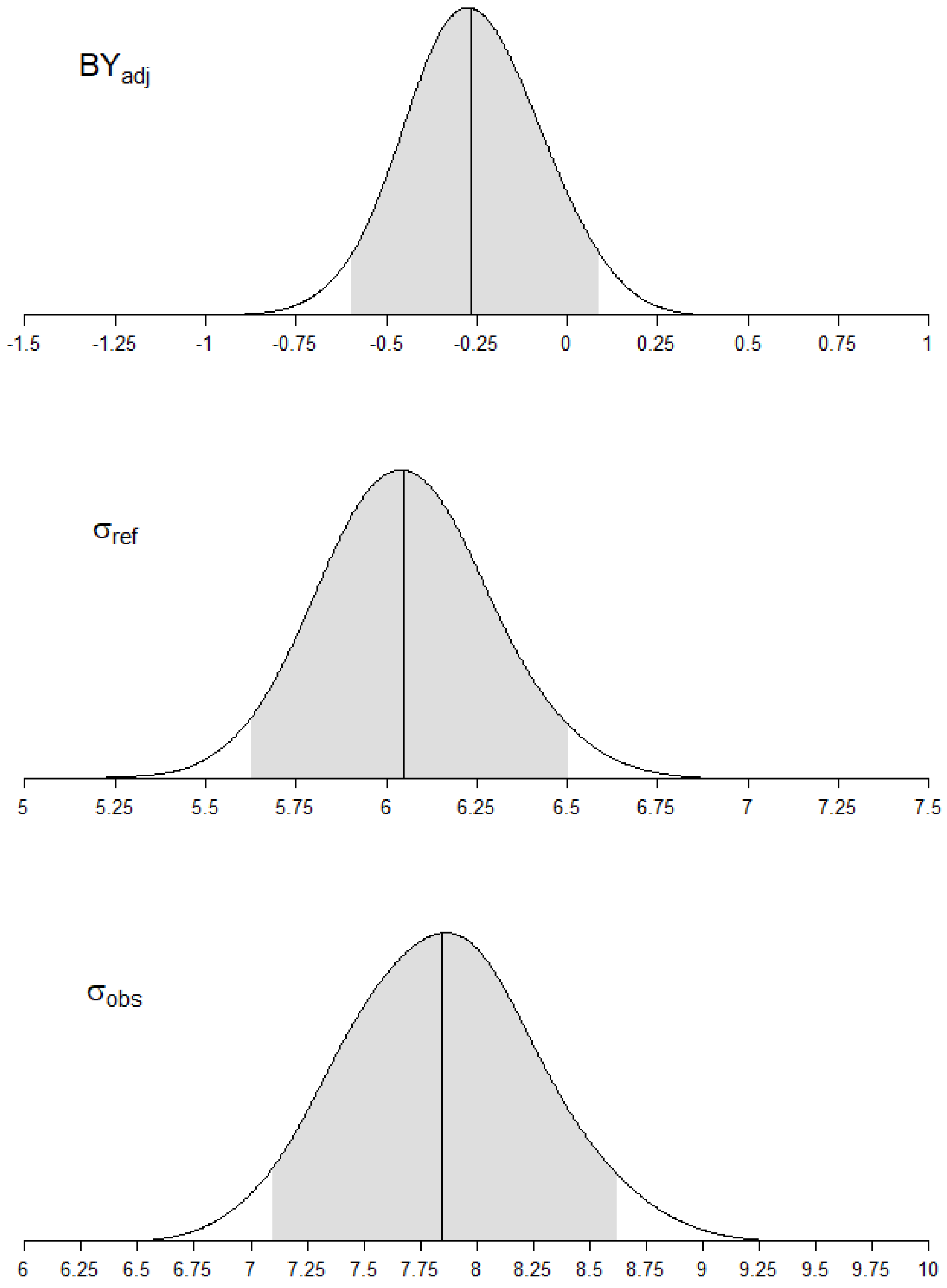

Figure S2. Posterior distributions of $\Delta^{14}C$ Bayesian spline model parameter estimates. The median (black solid line) and 95% credible intervals (CI; shaded area) where BY_{adj} is the ageing bias adjustment to the birthyear estimate, σ_{ref} is the standard deviation of the reference series observations around the expected values, and σ_{obs} is the standard deviation of the observed $\Delta^{14}C$ values around the expected.

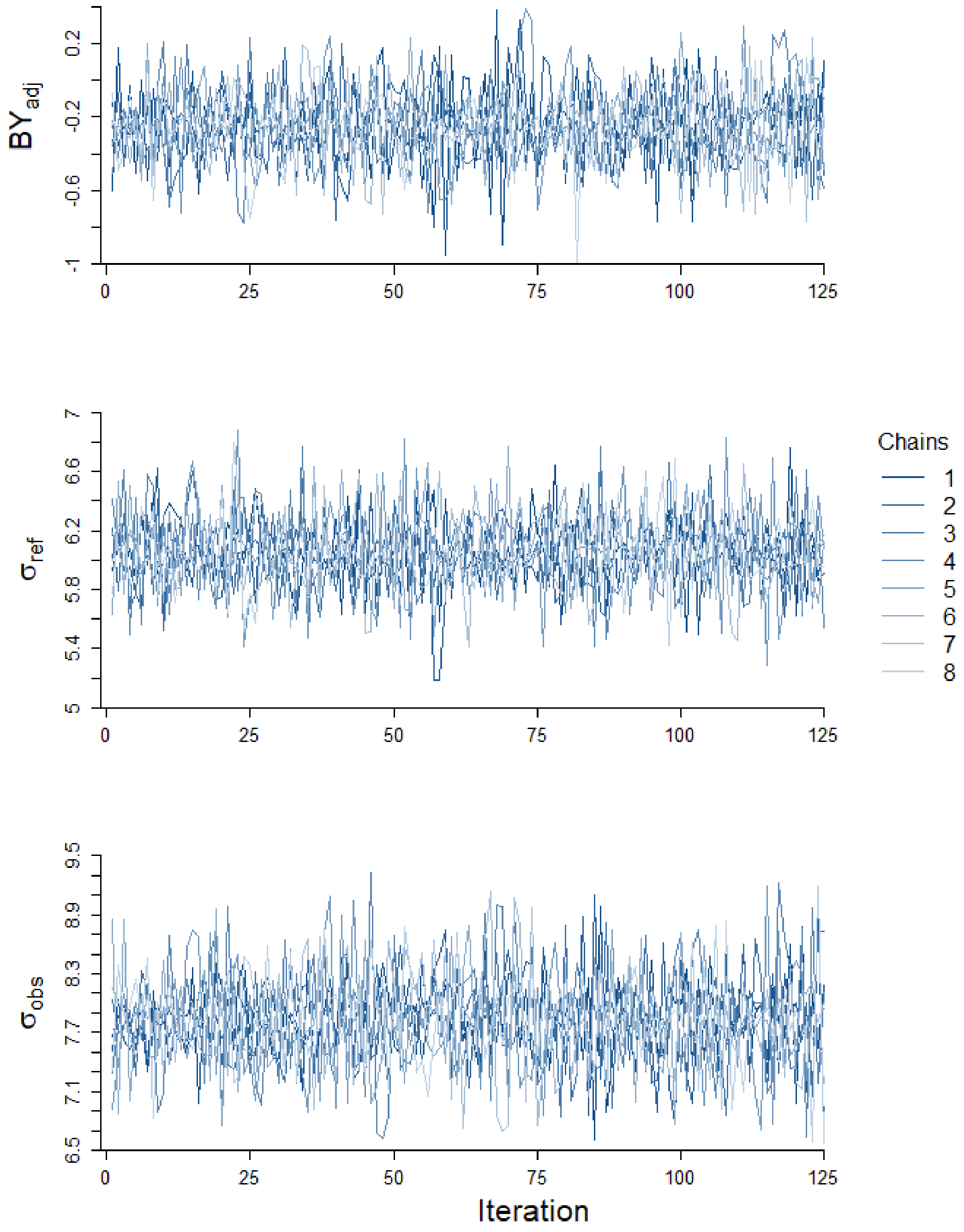

Figure S3. Chains of Δ^{14}C Bayesian spline model parameter estimates, where BY_{adj} is the ageing bias adjustment to the birthyear estimate, σ_{ref} is the standard deviation of the reference series observations around the expected values, and σ_{obs} is the standard deviation of the observed $\Delta^{14}C$ values around the expected.

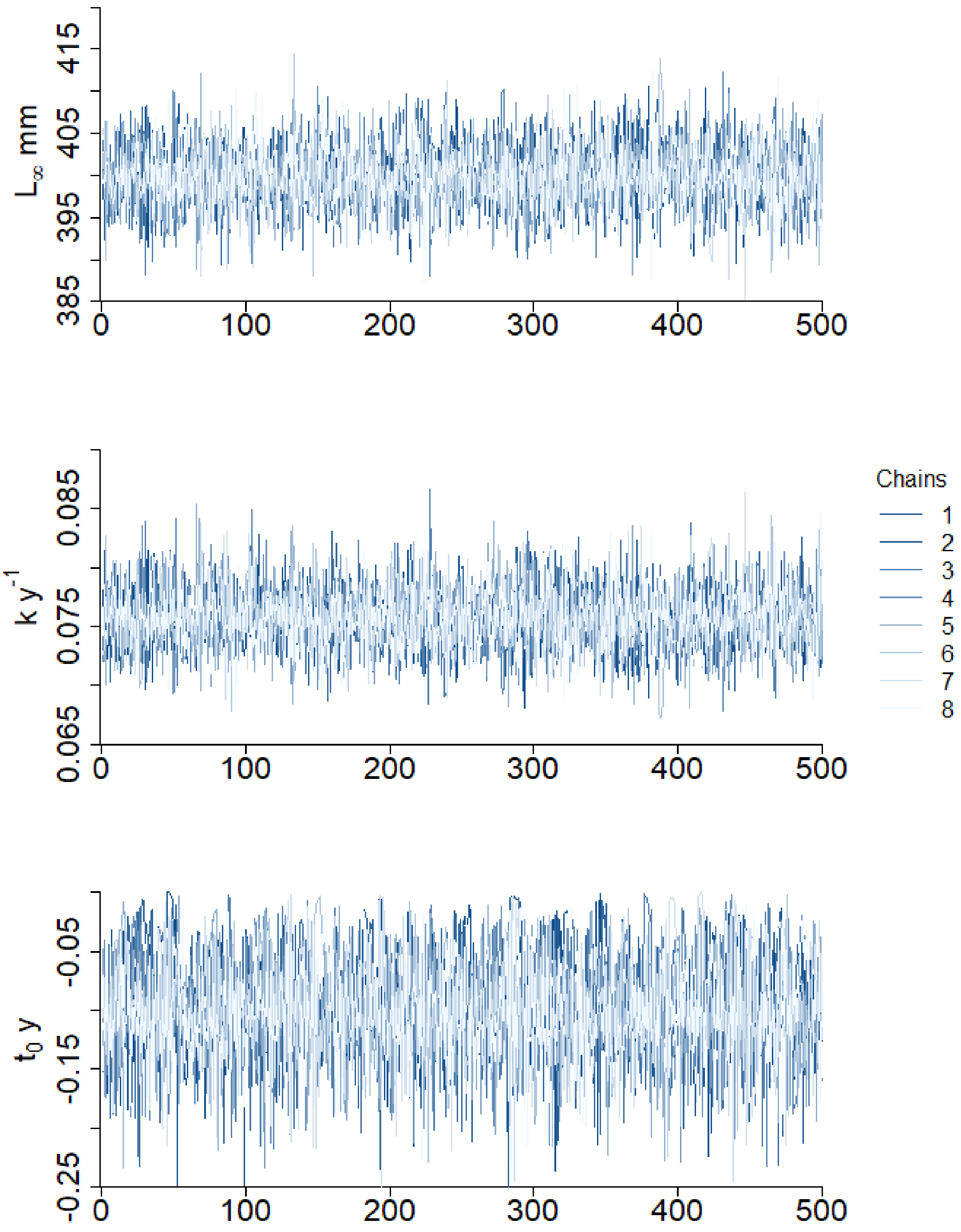

Figure S4. Chains of von Bertalanffy growth model parameter estimates, where L_∞ is asymptotic length, k is the Brody growth coefficient, and t_0 is the theoretical age at which length is 0.

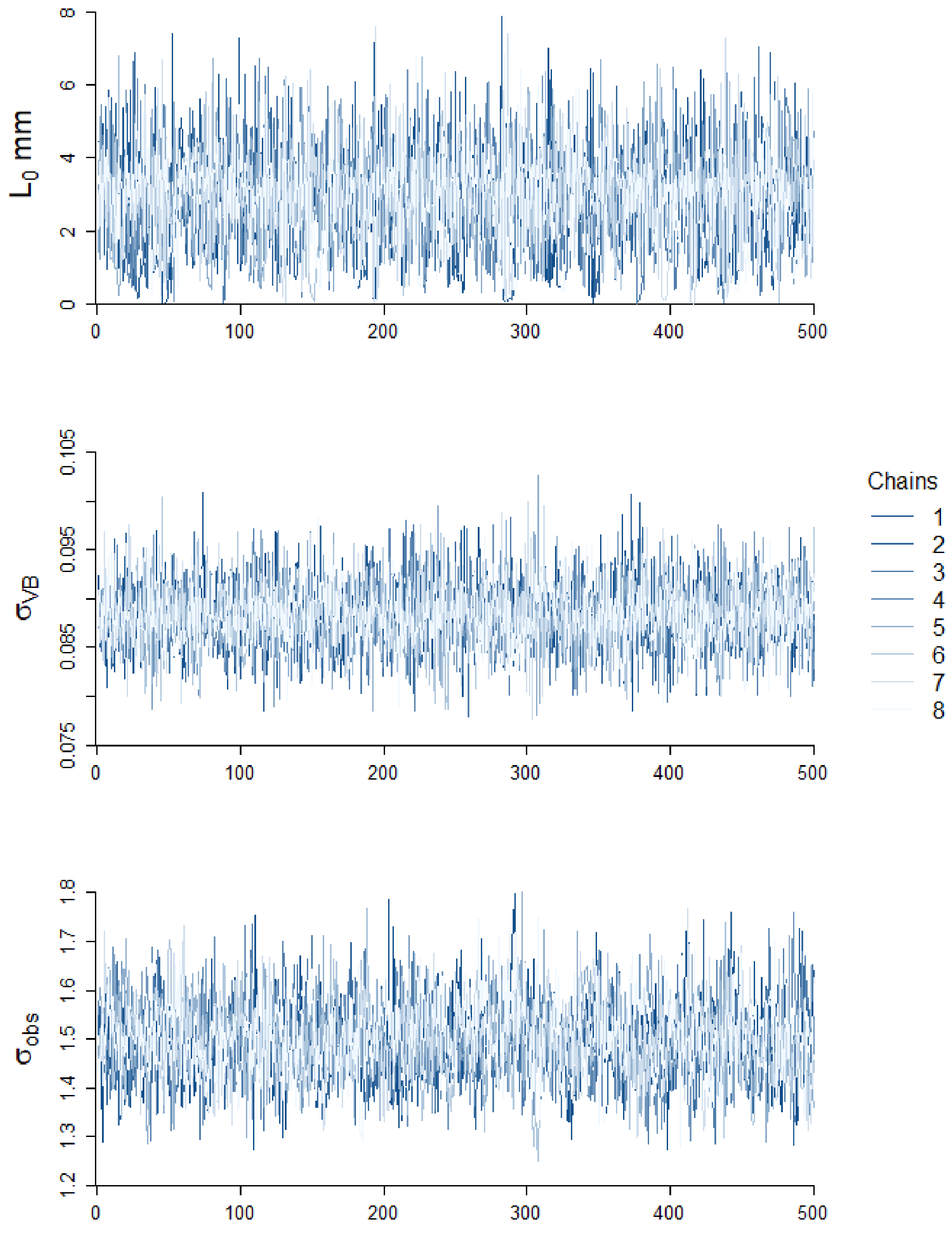

Figure S4 continued. Chains of von Bertalanffy growth model parameter estimates, where L_0 is length-at-birth, σ_{VB} is the likelihood standard deviation of the von Bertalanffy growth model (VBGM), and σ_{obs} is the measure of inter-reader error.
